# The NAD^+^ challenge: Harnessing immune-mediated tumor control without fueling the enemy

**DOI:** 10.1016/j.omton.2026.201311

**Published:** 2026-08-10

**Authors:** Eryn Zuiker, Emily A. Hemann, Matthew E. Long

**Affiliations:** 1 Department of Microbial Infection and Immunity, The Ohio State University Wexner Medical Center, Columbus, OH, USA; 2 Dorothy M. Davis Heart and Lung Research Institute, The Ohio State University Wexner Medical Center, Columbus, OH, USA

## Main text

In a recently published paper in *Molecular Therapy Oncology*, Xu et al.^1^ utilized a murine AB1 mesothelioma tumor model to test the ability of nicotinamide (NAM) mononucleotide (NMN) supplementation, a precursor to NAD^+^, to halt tumor progression and elicit effects on immune cell phenotypes in the tumor microenvironment (TME). The study demonstrated that mice treated with NMN exhibited tumor burden control equivalent to mice treated with an immune checkpoint blockade PD1 inhibitor. While α-PD1 treatment increased the number of CD8^+^ T cells and reduced monocytic myeloid-derived suppressor cells (M-MDSCs) and polymorphonuclear (PMN-MDSCs) in this model, NMN did not alter these immune cell populations. Instead, NMN treatment skewed macrophage polarization to increase CD206^−^ “M1” inflammatory macrophages while decreasing CD206^+^ “M2” anti-inflammatory macrophages. Depletion of macrophages significantly reduced the tumor-suppressing effects of NMN treatment, strengthening the link between NMN and modulation of macrophage anti-tumor functions. While advances in immunotherapies, including immune checkpoint blockade, have been transforming cancer treatment, their effectiveness varies widely, with ample room for improvement.^2^ New approaches to enhance the anti-tumor immune response are active areas of research and development. The ability of NMN to provide robust anti-tumor immune responses is intriguing, though specific mechanisms by which NAD^+^ supplementation methods may enhance immune cell function require further investigation, and the potential of NAD^+^ to fuel cancer cell growth or resistance to treatments needs rigorous in-depth evaluation.

The functions of NAD^+^ and the cellular and biochemical mechanisms regulating NAD^+^ metabolism have been studied since 1906.^3^ Major discoveries in the field have revealed that NAD^+^ biosynthesis occurs through three main pathways: the kynurenine pathway, which uses tryptophan as a starting source; the press-handler pathway, which uses nicotinic acid; or the salvage pathway, in which NMN is generated from either NAM or NAM riboside (NR) and serves as the precursor to NAD^+^.^4^ A rate-limiting step in the salvage pathway is the conversion of NAM to NMN by the enzyme NAM phosphoribosyltransferase (NAMPT); however, bypassing this step by providing NMN (or NR) supplementation allows for NMN conversion to NAD^+^ by one of three NMN adenylyltransferase (NMNAT) proteins encoded in humans.^4^ NAD^+^ declines and linkage to pathologies have been well documented across multiple tissues during aging, obesity, cardiovascular and neurological diseases, and cancer.^3^ Therefore, multiple strategies to increase NAD^+^ have been developed and are being evaluated as potential therapeutic treatments.

In the current study, human peripheral blood mononuclear cells (PBMCs) co-cultured with A549 cells were treated with increasing doses of NMN, which accumulated in the PBMCs and increased the expression of NMNAT1, NMNAT2, and NMNAT3, although specific functional results from this treatment were not interrogated. To interrogate the effects of NMN in an *in vivo* model, AB1 engrafted mice were intraperitoneally (i.p.) treated with vehicle, 200 mg/kg α-PD1, or 300 mg/kg NMN at the time of tumor inoculation and twice weekly through day 14. Over the 2-week study, mice treated with α-PD1 and NMN both had reduced tumor burdens of the same magnitude. While α-PD1 treatment significantly increased the number of tumor-infiltrating CD8 T cells and reduced M-MDSCs and PMN-MDSCs, NMN treatment elicited no changes in CD8 T cells, NK cells, M-MDSCs, or PMN-MDSCs compared to vehicle-treated mice. However, NMN treatment significantly increased CD206^−^ macrophages while reducing CD206^+^ macrophages, indicating a skewing of macrophage phenotypes toward increased so-called M1 polarization and decreased M2 polarization phenotypes. In separate experiments, depletion of macrophages with an anti-CD115 (CSF1R) antibody 2 days prior to tumor implantation, followed by NMN treatment, resulted in decreased control of tumor burden compared to NMN treatment alone, suggesting that a substantial portion of the anti-tumor effect of NMN is macrophage dependent.

Splenic macrophages or CD8 T cells isolated from AB1 engrafted mice and *ex vivo* co-cultured with AB1 cells either in the absence or presence of NMN for 24 h revealed that macrophage co-cultures with NMN had significantly increased *Tnf*, *Il12b*, and *Cd38* expression. In contrast, only *Cd38* was significantly increased in CD8 T cells following NMN. To validate these findings *in vivo*, CD11b^+^F4/80^+^ macrophages and CD8 T cells were examined on day 14, confirming that TNF, IL-12β, CD38, and IFNγ were significantly increased in CD11b^+^F4/80^+^ macrophages in the NMN-treated group. In CD8 T cells, levels of these proteins were not altered following NMN treatment. Overall, these results support the notion that NMN elicits changes in macrophage polarization, though the functional outcomes of these alterations remain unclear. For example, CD38 hydrolyzes NAD^+^, and increased CD38 activity results in decreased intracellular and tissue NAD^+^ concentrations, whereas blocking CD38 enzymatic activity can increase NAD^+^.^5^ Therefore, increased macrophage CD38 may indicate decreased availability of NAD^+^ and should be further investigated. Additional functional and mechanistic links between NAD^+^ levels and immune cell activation, including macrophages, have previously been recognized but need further exploration in models of the TME.^6^

Previous studies investigated the effects of NAD^+^ precursor supplementation on immune function during cancer therapy. For example, the decreased tumor-killing capacity of T cells in the TME was in part due to decreased *NAMPT* expression in tumor-infiltrating lymphocytes (TILs), resulting in decreased intracellular NAD^+^ and impaired anti-tumor functions.^7^ Interestingly, this study demonstrated that daily 500 mg/kg i.p. supplementation of NAM increased the tumor-killing ability of CAR-T cells. This study also demonstrated that while NAM and α-PD1 each elicited individual anti-tumor effects, combined NAM and α-PD1 treatments had additive beneficial effects. Although macrophage phenotypes were not examined in the previous study, the phenotypes reported by Xu et al. suggest they may be worth investigating. In contrast to potential benefits of increasing NAD^+^ levels, other research has indicated that cancer cells utilize NAD^+^ for survival. In fact, previous studies have demonstrated the benefits of NAMPT pharmacological inhibitors in targeting cancer cells, although the pursuit of clinical therapeutics in this area has been hampered by the overall toxicity elicited by NAMPT inhibitors.^8^ Overall, a major concern in attempts to increase NAD^+^ is the potential for NAD^+^ to fuel cancer cell growth or survival. Further highlighting potential unwanted effects of NAD^+^-boosting therapies, a recent study using pancreatic ductal adenocarcinoma models demonstrated that NAD^+^ precursors, including NMN, increased chemotherapy resistance *in vitro* and *in vivo*.^9^

**Figure 1:**
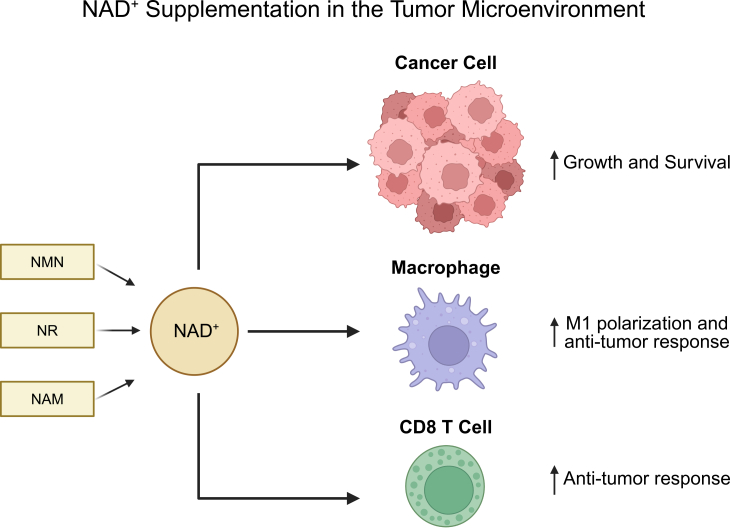
Therapeutic potential of NAD^+^ supplementation in the tumor microenvironment Different strategies to increase NAD^+^ in the tumor microenvironment through supplementation with nicotinamide mononucleotide (NMN), nicotinamide riboside (NR), or nicotinamide (NAM) may enhance macrophage and CD8 T cell anti-tumor immune responses but may also elicit detrimental impacts by increasing cancer cell growth and survival. Figure created in BioRender. Long, M. (2026) https://BioRender.com/buoaut5.

It is interesting to speculate that increasing NAD^+^ may be a ripe area for modulating the immune response in cancer therapy. However, additional studies should perform direct comparisons of NAM, NMN, and NR supplementation in combination with α-PD1 therapies to provide a more comprehensive assessment of the therapeutic potential of these treatments. Individual cancer types should also be evaluated for contraindications to these strategies to boost NAD^+^. Studies that better model likely delivery to human patients should also be incorporated, as many NAD^+^-boosting strategies rely on oral delivery where interactions with the gut microbiome may affect metabolism and NAD^+^ levels.^10^ How NAD^+^ boosting strategies may alter the anti-tumor immune response when administered later in disease, rather than at the time of tumor cell engraftment, should also be assessed in animal models, and determining whether NMN, NR, or NAM modulate macrophage phenotypes in other tumor models would increase the generalizability of the results (Figure 1).

## Declaration of interests

The authors declare no competing interests.
